# Supplementary figures and images for: A Sir2-regulated locus control region in the recombination enhancer of Saccharomyces cerevisiae specifies chromosome III structure
Source: PLoS Genet. 2019 Aug 28;15(8):e1008339. doi: 10.1371/journal.pgen.1008339 (PMC6736312; doi:10.1371/journal.pgen.1008339)

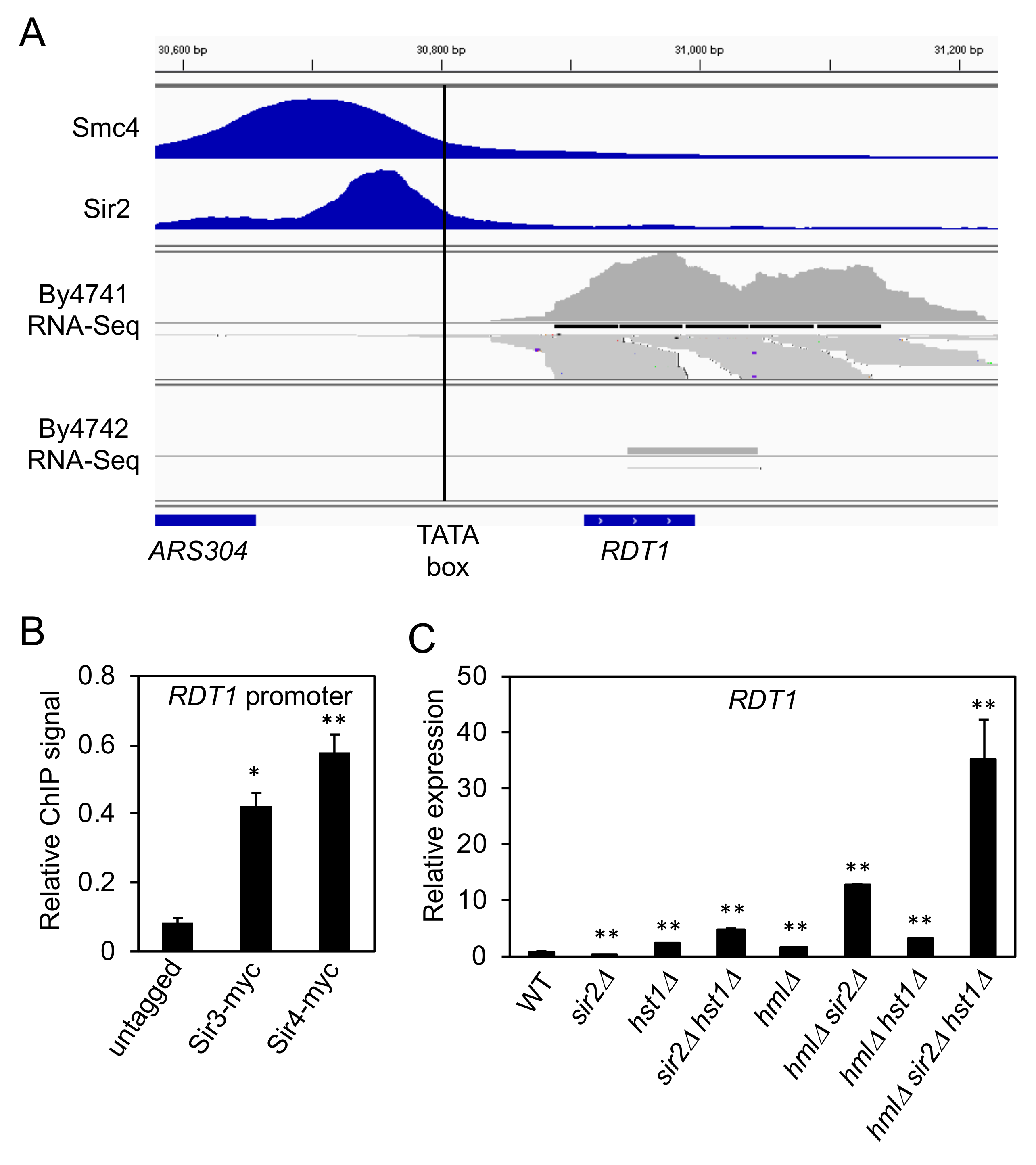

Supplement: S1 Fig — (A) IGV screenshot of compiled raw RNA-seq read data from BY4741 (MATa) and BY4742 (MATα) strains. The top two blue peaks represent Smc4-myc and Sir2-myc ChIP-seq reads. (B) Quantitative ChIP assay showing additional SIR complex subunit enrichment at the RDT1 promoter. Signals are relative to input. (C) RT-qPCR showing effects of deleting SIR2 and/or HST1 on RDT1 expression (relative to ACT1) when HML is present or deleted (*p<0.05, **p < .005). The WT RDT1/ACT1 ratio is normalized to 1. (TIF) [file pgen.1008339.s001.tif]

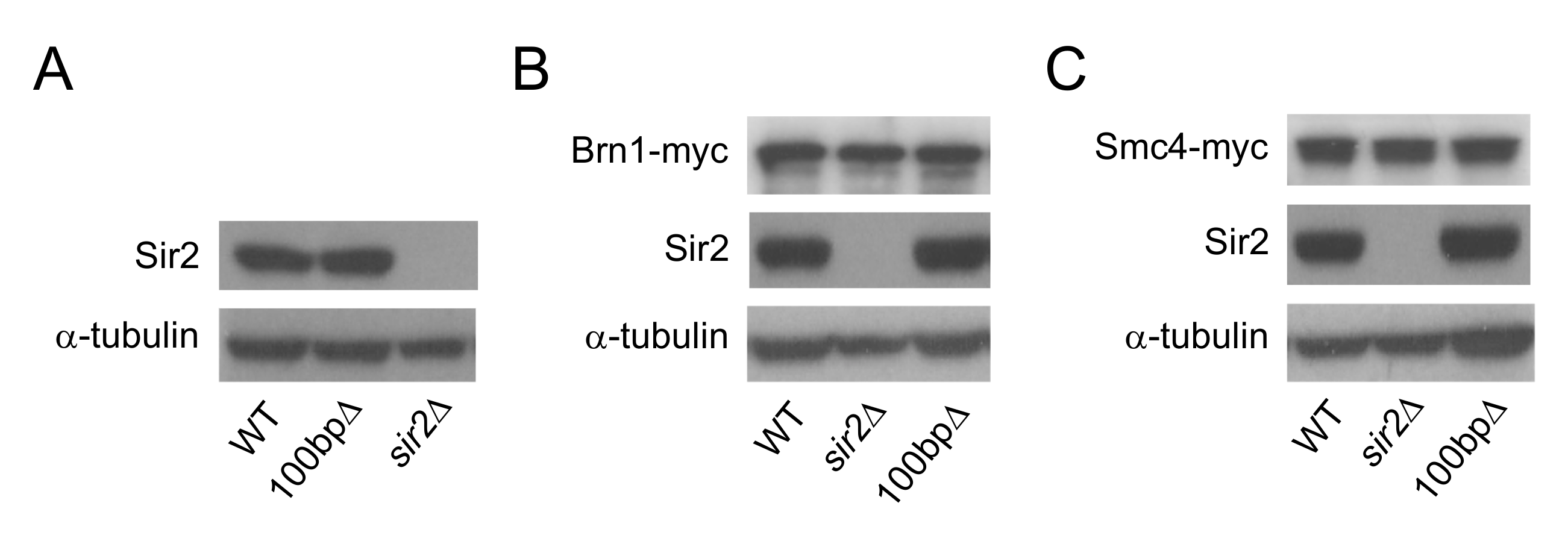

Supplement: S2 Fig — (A) Western blot showing steady state Sir2 protein levels in WT (ML1), sir2Δ (ML25), and 100bpΔ (ML275) strains. (B) Western blot with anti-Myc detection of Brn1-13xMyc or Sir2 in WT (ML149), sir2Δ (ML161), and 100bpΔ (ML322) strains. (C) Western blot with anti-Myc detection of Smc4-13xMyc or Sir2 in WT (ML152), sir2Δ (ML160), and 100bpΔ version. (TIF) [file pgen.1008339.s002.tif]

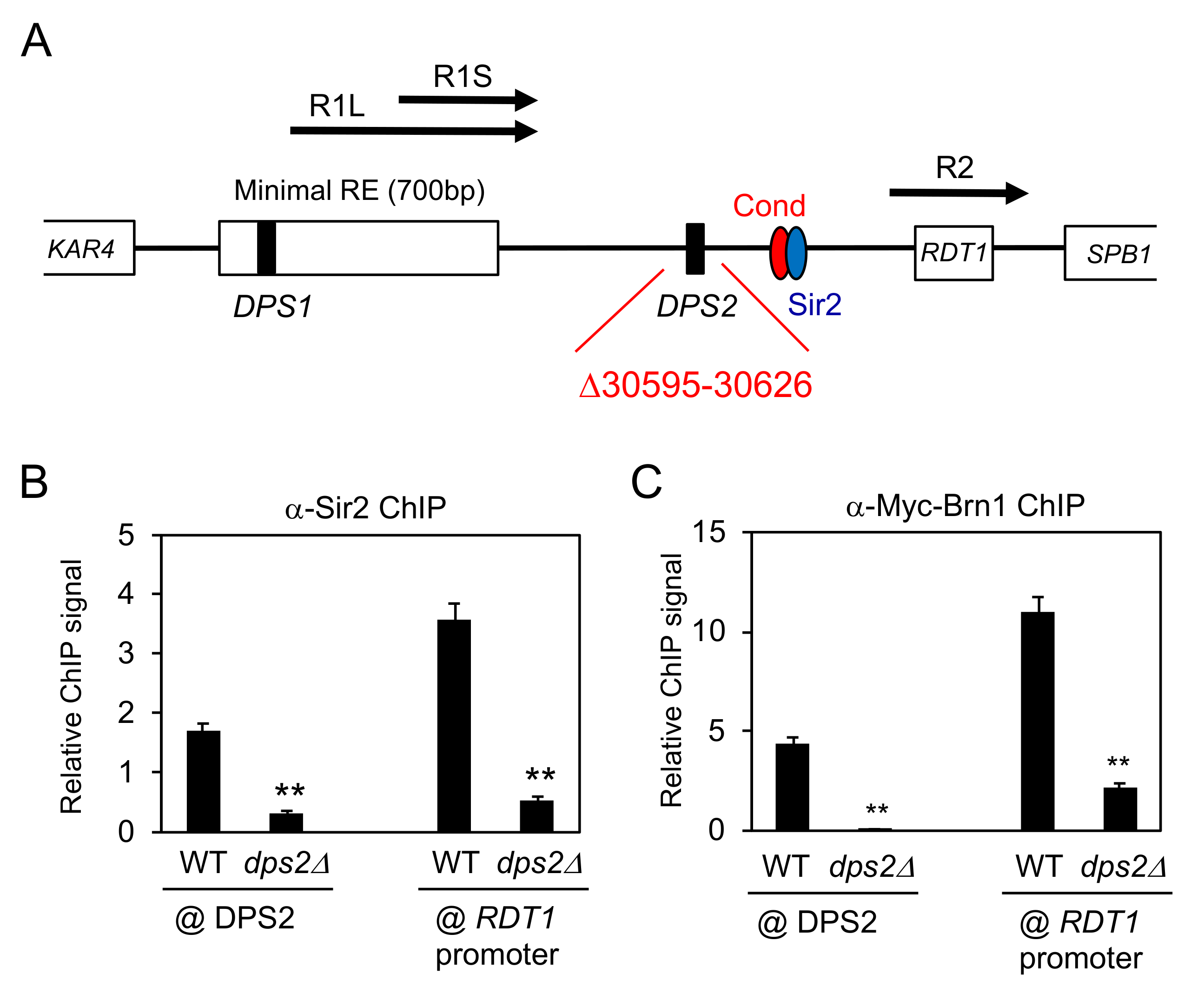

Supplement: S3 Fig — (A) Schematic diagram depicting the location of the DPS2 sequence deletion relative to other elements with the RE, with the deleted chromosome III coordinates indicated in red. (B) Quantitative ChIP of native Sir2 in WT and dps2Δ strains. (C) Quantitative ChIP of Brn1-Myc in WT and dps2Δ strains. @RDT1 promoter indicates enrichment at the Sir2/condensin peak. ChIP signals are plotted relative to the input signal. (**p<0.005). (TIF) [file pgen.1008339.s003.tif]

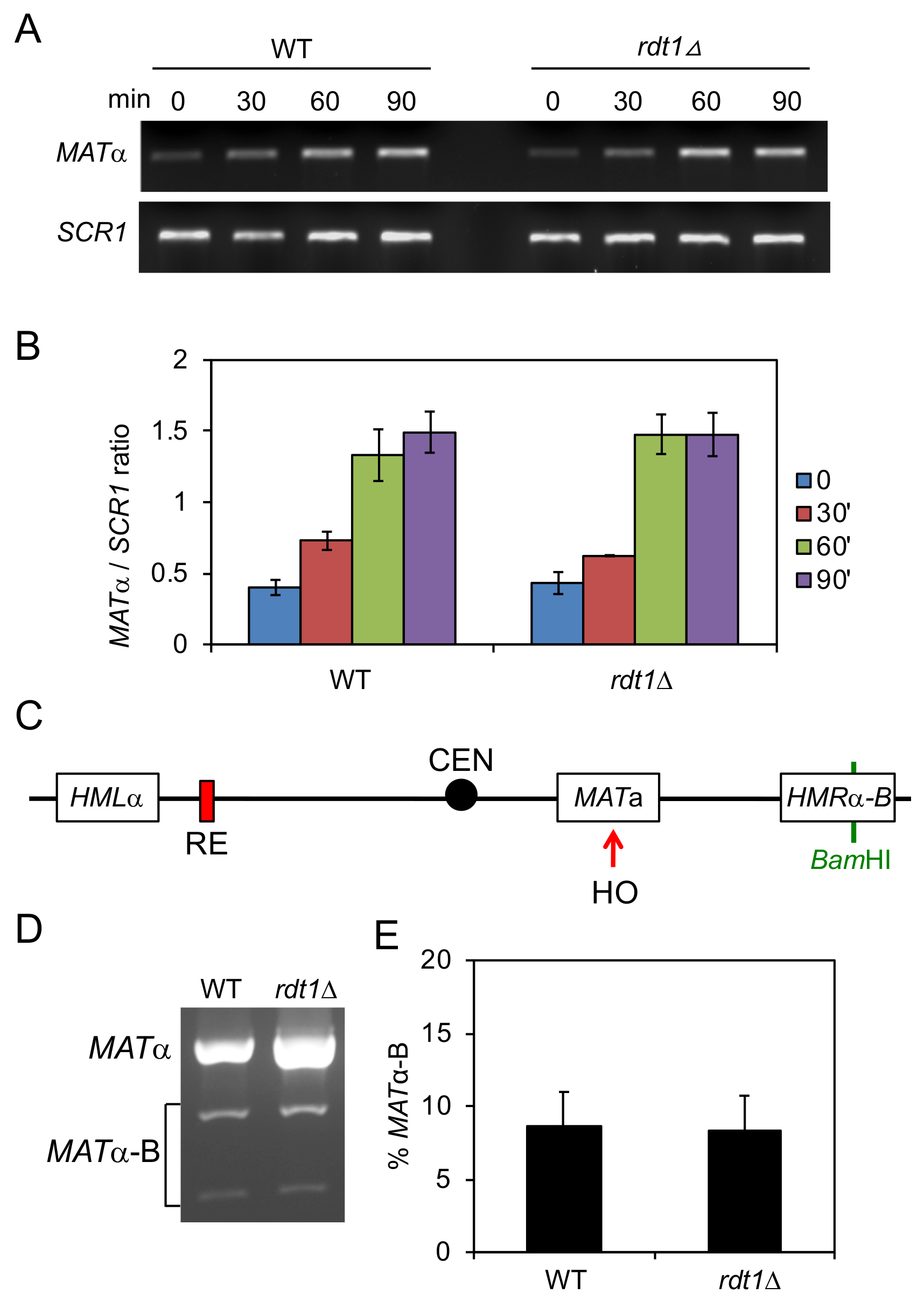

Supplement: S4 Fig — (A) Representative time course of switching from MATa to MATα in WT (ML440) and rdt1Δ (ML443) strains. PCR products are specific to MATα and an SCR1 loading control. (B) Quantitation of the average MATα/SCR1 PCR signal ratio from 3 biological replicates. (C) Schematic of chromosome III in the donor preference reporter strain harboring an artificial HMRα-B cassette as the donor for switching, which introduces a unique BamHI site to the MAT locus. (D) Representative ethidium bromide stained agarose gel of BamHI-digested MATα PCR products after mating-type switching in WT (XW652) and rdt1Δ (MD30) strains. The bottom two bands represent HMR-derived switching. (E) Quantitation of average MATα-B utilization from 3 biological replicates. (TIF) [file pgen.1008339.s004.tif]

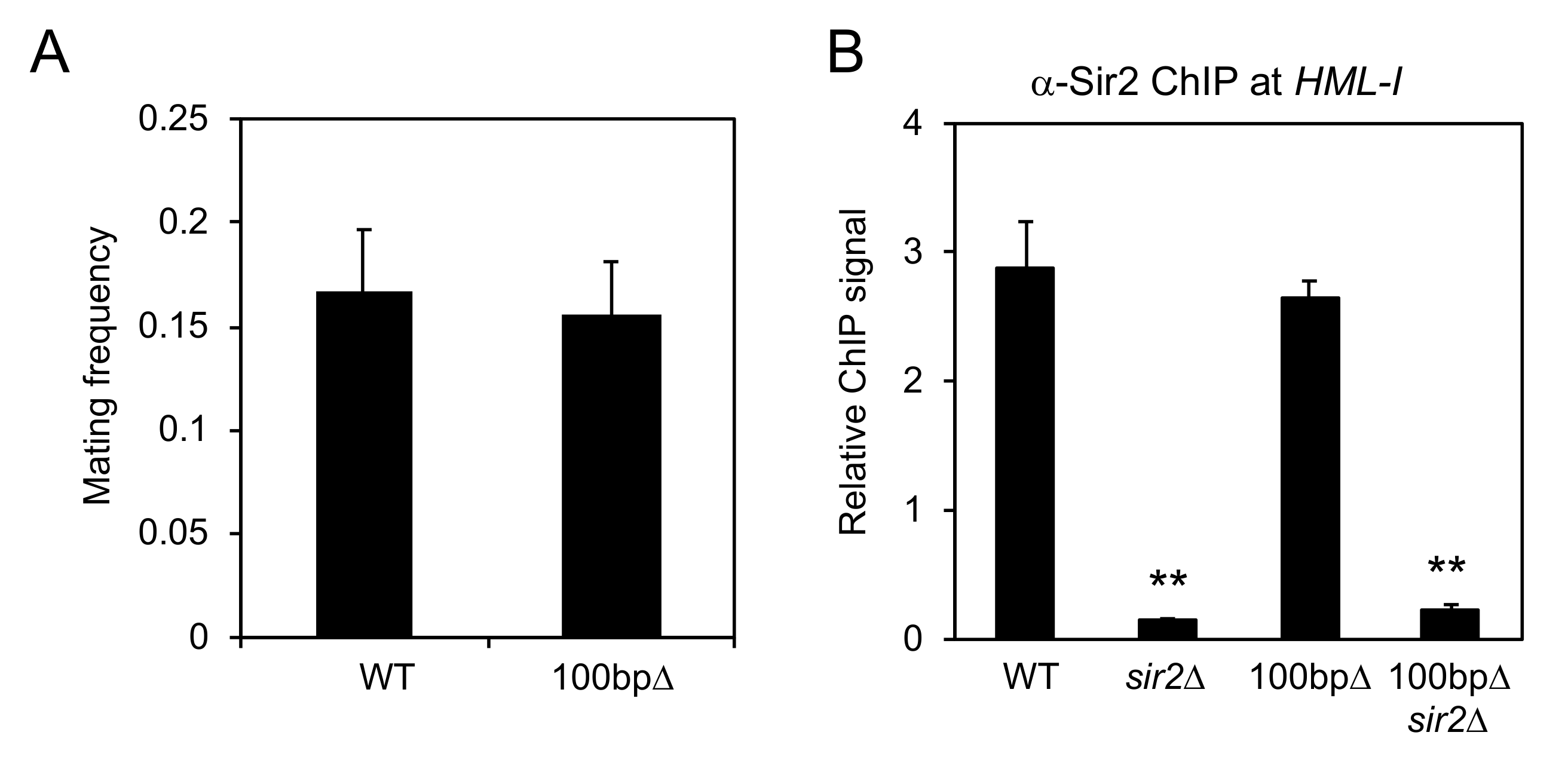

Supplement: S5 Fig — (A) Quantitative mating assay for WT (ML1) and 100bpΔ (ML275) strains. (B) Quantitative ChIP assay showing Sir2 enrichment at HML-I in WT (ML1) and 100bpΔ (ML275) strains. (**p<0.005). (TIF) [file pgen.1008339.s005.tif]

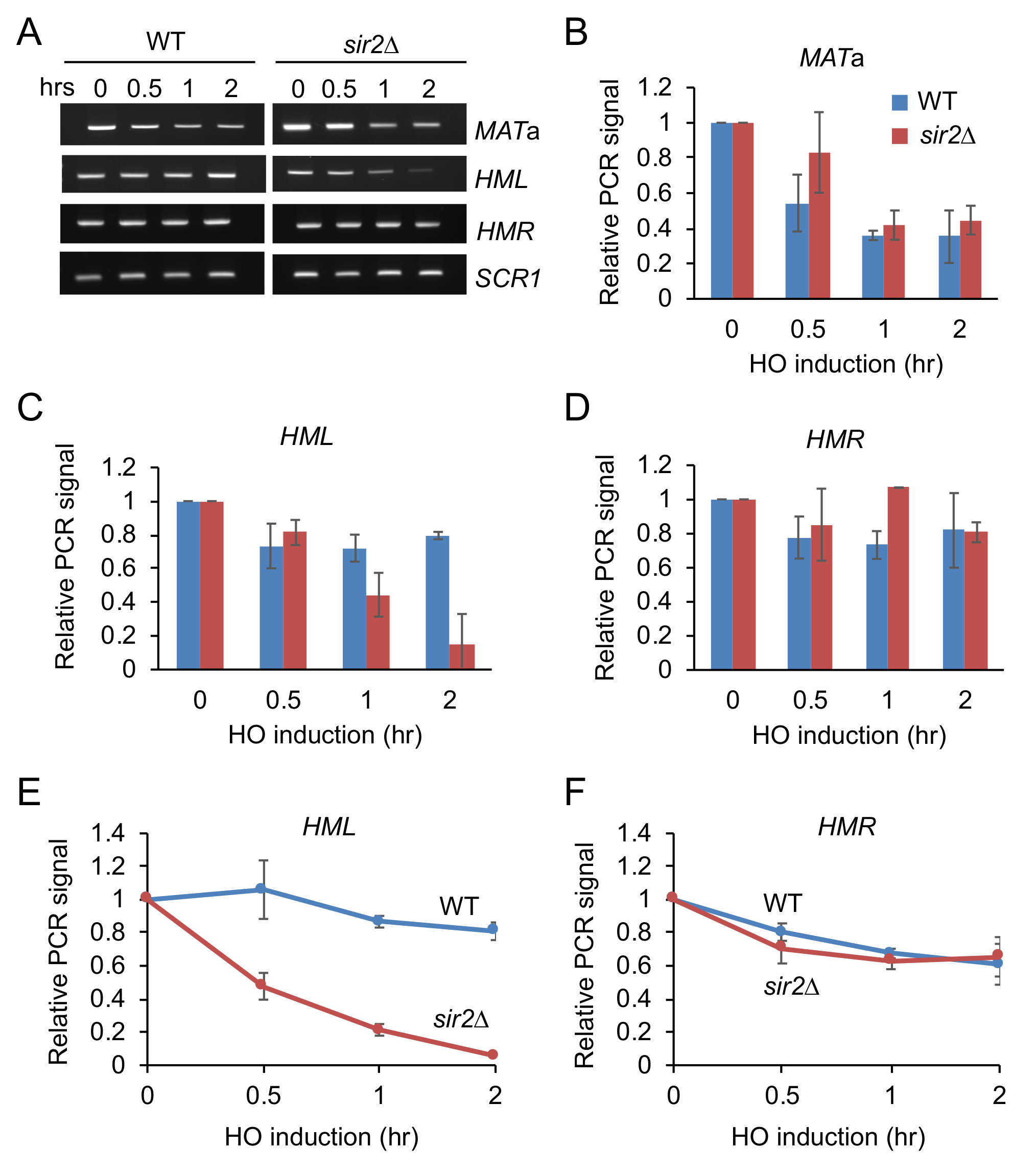

Supplement: S6 Fig — (A) PCR amplification of MATa, HML, HMR, and SCR1 loci from MATa WT (ML440) and sir2Δ (MD29) strains containing the pGAL-HO-URA3 expression vector. Times after HO induction with galactose are indicated. A representative experiment is depicted. (B) Quantitation of mean MATa PCR signal, relative to the SCR1 control, from 3 independent biological replicates. Error bars indicate standard deviation. (C) Quantitation of mean HML PCR signal, as done for panel B. (D) Quantitation of mean HMR PCR signal, as done for panel B. (E and F) Real-time qPCR signal, relative to SCR1 control, for HML and HMR, respectively. The PCR signal at time 0 is normalized to 1.0 in each panel. (TIF) [file pgen.1008339.s006.tif]

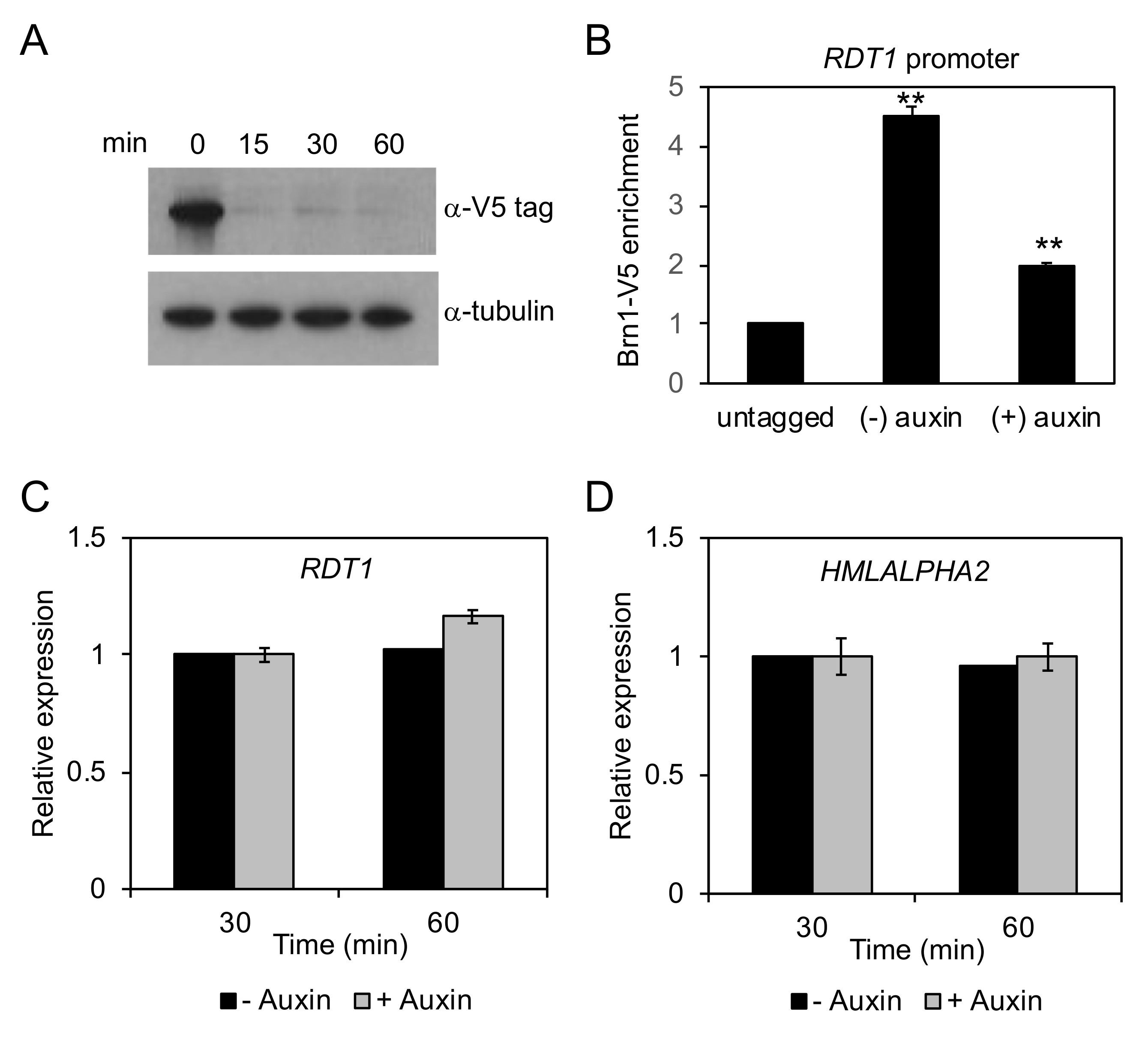

Supplement: S7 Fig — (A) Western blot time course of auxin induced degradation of Brn1-V5. Time indicates minutes after addition of auxin. (B) ChIP assay showing Brn1-V5 enrichment at the RDT1 promoter with and without auxin addition. The Brn1-V5 signal is relative to input and the ratio from an untagged control strain is normalized to 1. (C) RT-qPCR of RDT1 expression following 30 or 60 minutes of Brn1 depletion by auxin. (D) RT-qPCR of HMLALPHA2 expression following 30 or 60 min of Brn1 depletion by auxin. In panels C and D, the signals are relative to ACT1 control and normalized to 1.0 without auxin. (TIF) [file pgen.1008339.s007.tif]
