# Supplementary material for: A Sir2-regulated locus control region in the recombination enhancer of Saccharomyces cerevisiae specifies chromosome III structure
Source: PLoS Genet. 2019 Aug 28;15(8):e1008339. doi: 10.1371/journal.pgen.1008339 (PMC6736312; doi:10.1371/journal.pgen.1008339)
Supplement: S2 Table — List of all Saccharomyces cerevisiae strains used in this study, along with their genotypes and source. (DOCX) [file pgen.1008339.s009.docx]

Supplemental Table S2. Yeast Strains

| **Strains** | **Genotype** | **Source** |
| --- | --- | --- |
| ML1 | *MAT*a *his3∆200 leu2∆1 met15∆0 trp1∆63 ura3-167* | [1] |
| ML25B12 | *MAT*α *his3∆200 leu2∆1 met15∆0 trp1∆63 ura3-167* | This study |
| ML25 | *MAT*a *his3∆200 leu2∆1 met15∆0 trp1∆63 ura3-167 sir2∆::kanMX4* | [1] |
| ML26 | *MAT*a *his3∆200 leu2∆1 met15∆0 trp1∆63 ura3-167 sir3∆::kanMX4* | [1] |
| ML27 | *MAT*a *his3∆200 leu2∆1 met15∆0 trp1∆63 ura3-167 sir4∆::kanMX4* | [1] |
| ML28 | *MAT*α *his3∆200 leu2∆1 met15∆0 trp1∆63 ura3-167 sir2∆::kanMX4* | This study |
| ML149 | *MAT*a *his3∆200 leu2∆1 met15∆0 trp1∆63 ura3-167*  *BRN1::13xMyc-kanMX4* | [2] |
| ML152 | *MAT*a *his3∆200 leu2∆1 met15∆0 trp1∆63 ura3-167*  *SMC4::13xMyc-kanMX4* | [2] |
| ML160 | *MAT*a *his3∆200 leu2∆1 met15∆0 trp1∆63 ura3-167*  *SMC4::13xMyc-kanMX4 sir2Δ::natMX4* | [2] |
| ML161 | *MAT*a *his3∆200 leu2∆1 met15∆0 trp1∆63 ura3-167*  *BRN1::13xMyc-kanMX4 sir2Δ::natMX4* | [2] |
| ML195 | *MAT*a *his3∆200 leu2∆1 met15∆0 trp1∆63 ura3-167 sir2∆::kanMX4* [pRS315] | This study |
| ML196 | *MAT*a *his3∆200 leu2∆1 met15∆0 trp1∆63 ura3-167 sir2∆::kanMX4* [pRS315-*SIR2*] | This study |
| ML197 | *MAT*a *his3∆200 leu2∆1 met15∆0 trp1∆63 ura3-167 sir2∆::kanMX4* [pRS315-*sir2-H364Y*] | This study |
| ML275 | ML1 deleted for the 100bp Sir2/condensin binding site (100bp∆) | This study |
| ML279 | ML275 made *sir2∆::kanMX4* | This study |
| ML286 | ML25B12 deleted for the 100bp Sir2/condensin binding site (100bp∆) | This study |
| ML322 | ML149 deleted for the 100bp Sir2/condensin binding site (100bp∆) | This study |
| ML337 | *MAT*α *his3∆200 leu2∆1 met15∆0 trp1∆63 ura3-167 BRN1::13xMyc-kanMX4* | This study |
| ML339 | *MAT*α *his3∆200 leu2∆1 met15∆0 trp1∆63 ura3-167 SMC4::13xMyc-kanMX4* | This study |
| ML341 | *MAT*a *his3∆200 leu2∆1 met15∆0 trp1∆63 ura3-167 dps2∆* | This study |
| ML342 | *MAT*a *his3∆200 leu2∆1 met15∆0 trp1∆63 ura3-167 rdt1∆* | This study |
| ML343 | *MAT*a *his3∆200 leu2∆1 met15∆0 trp1∆63 ura3-167 sir2∆::kanMX4 hml∆::LEU2* | [1] |
| ML344 | *MAT*a *his3∆200 leu2∆1 met15∆0 trp1∆63 ura3-167 hml∆::LEU2* | [1] |
| ML350 | *MAT*a *his3∆200 leu2∆1 met15∆0 trp1∆63 ura3-167 BRN1::13xMyc-kanMX4 hml∆::LEU2* | This study |
| ML351 | *MAT*a *his3∆200 leu2∆1 met15∆0 trp1∆63 ura3-167 BRN1::13xMyc-kanMX4 sir2∆::kanMX4 hml∆::LEU2* | This study |
| ML419 | *MAT*a *his3∆200 leu2∆1 met15∆0 trp1∆63 ura3-167*  *RDT1::13xMyc-kanMX4* | This study |
| ML432 | ML419 deleted for the 100bp Sir2/condensin binding site (100bp∆) | This study |
| ML433 | *MAT*α *his3∆200 leu2∆1 met15∆0 trp1∆63 ura3-167*  *RDT1::13xMyc-kanMX4* | This study |
| ML440 | ML1 [pGAL-HO-*URA3*] | This study |
| ML443 | ML342 [pGAL-HO-URA3] | This study |
| ML444 | ML149 [pGAL-HO-*URA3*] | This study |
| ML523 | *MAT*a *his3∆200 leu2∆1 met15∆0 trp1∆63 ura3-167*  *BRN1::13xMyc-kanMX4 dps2∆* | This study |
| ML557 | XW652 *sir2∆::kanMX4* | This study |
| XW652 | *MAT***a** *ho ade3::GAL::HO HML*α*RE HMR*α-B *ura3-52 lys5 leu2-3,112 trp1::hisG* | [3] |
| XW676 | *MAT***a** *ho ade3::GAL::HO HML*α*RE∆::URA3 HMR*α-B *ade1 leu2 trp1 ura3-52* | [3] |
| SY742 | XW652 deleted for the 100bp Sir2/condensin binding site (100bp∆) | This study |
| NBY8 | *MATa ura3-1 leu2-3,112 his3-11 trp1-1 ade2-1 can1-100 bar1∆ lys2∆* | [4] |
| NBY316 | *MATa ura3-1 leu2-3,112 his3-11 trp1-1 ade2-1 can1-100 bar1∆ lys2∆ ycs4-1* | [4] |
| NBY585 | *MATa ura3-1 leu2-3,112 his3-11 trp1-1 ade2-1 can1-100 bar1∆ lys2∆ hml∆::LEU2* | [4] |
| NBY319 | *MATa ura3-1 leu2-3,112 his3-11 trp1-1 ade2-1 can1-100 bar1∆ lys2∆ hml∆::LEU2 ycs4-1* | [4] |
| RF15 | *MAT*a *his3∆200 leu2∆1 met15∆0 trp1∆63 ura3-167* [pRPL25NLS-GFP] | This study |
| RF25 | *MAT*a *his3∆200 leu2∆1 met15∆0 trp1∆63 ura3-167 sir2∆::kanMX4* [pRPL25NLS-GFP] | This study |
| MD25 | *MAT*a *leu2∆1 met15∆0 trp1∆63 his3∆200::pGPD1-Os TIR-HIS3 BRN1-3V5-AID2:KanMx6* [pGAL-HO-*URA3*] | This study |
| MD27 | XW652 made *leu2∆1::pGPD1-Os TIR-LEU2 BRN1::3V5-AID2-KanMX6* | This study |
| MD29 | ML25 [pGAL-HO-*URA3*] | This study |
| MD30 | XW652 made *rdt1∆* | This study |

1. Li M, Petteys BJ, McClure JM, Valsakumar V, Bekiranov S, Frank EL, et al. Thiamine biosynthesis in *Saccharomyces cerevisiae* is regulated by the NAD^+^-dependent histone deacetylase Hst1. Mol Cell Biol. 2010;30(13):3329-41. PMID: 20439498.

2. Li M, Valsakumar V, Poorey K, Bekiranov S, Smith JS. Genome-wide analysis of functional sirtuin chromatin targets in yeast. Genome Biol. 2013;14(5):R48. PMID: 23710766.

3. Li J, Coic E, Lee K, Lee CS, Kim JA, Wu Q, et al. Regulation of budding yeast mating-type switching donor preference by the FHA domain of Fkh1. PLoS Genet. 2012;8(4):e1002630. PMID: 22496671.

4. Bhalla N, Biggins S, Murray AW. Mutation of YCS4, a budding yeast condensin subunit, affects mitotic and nonmitotic chromosome behavior. Mol Biol Cell. 2002;13(2):632-45. PMID: 11854418.
