# Supplementary material for: A Sir2-regulated locus control region in the recombination enhancer of Saccharomyces cerevisiae specifies chromosome III structure
Source: PLoS Genet. 2019 Aug 28;15(8):e1008339. doi: 10.1371/journal.pgen.1008339 (PMC6736312; doi:10.1371/journal.pgen.1008339)
Supplement: S3 Table — List of oligodeoxynucleotides used in this study. (DOCX) [file pgen.1008339.s010.docx]

Supplemental Table S3. Oligonucleotides

| Oligo name | Oligo description | DNA sequence |
| --- | --- | --- |
| JS301 | *MAT*a or α PCR primer (FW) | AGTCACATCAAGATCGTTTATGG |
| JS302 | *MAT*α specific PCR primer (RV) | GCACGGAATATGGGACTACTTCG |
| JS467 | *KanMX* 5’-out detection | TACGGGCGACAGTCACATCATG |
| JS854 | *MAT*a specific PCR primer (RV) | ACTCCACTTCAAGTAAGAGTTTC |
| JS1830 | *SCR1* PCR primer (FW) | AATGGCTTTCTGGTGGGATG |
| JS1831 | *SCR1* PCR primer (RV) | TTGTTCCTCGGCCAGAATTC |
| JS1896 | *SPB1* ORF (FW) | CATCGAAGTTAAGGACGACGC |
| JS1897 | *SPB1* ORF (RV) | TCGCGCTTGACATTTAGACG |
| JS1898 | Sir2 binding site 100 bp (FW) | TGTTTGCAAGATGGTGCTTTTT |
| JS1899 | Sir2 binding site 100 bp (RV) | AGGAGCAGAAACGTGGCAAT |
| JS1909 | *HML-I* silencer (ARS302) (FW) | AACATGAAAGCCCGACGTTT |
| JS1910 | *HML-I* silencer (ARS302) (RV) | AATAATCGGGTGAAAAAGAGGATAT |
| JS2127 | *BRN1*_Degron_FW | AGTGAATTATGAGGATCTAGCGACAACACAGGCAGCGTCACGGATCCCCGGGTTAATTAA |
| JS2128 | *BRN1*_Degron_RV | GCACAAAAAAAAAAAAAAAAAAAAAAAAAAAGATCATCAAGAATTCGAGCTCGTTTAAAC |
| JS2167 | 3C *PDC1* intergenic FWD | GCCGACAGTCTGTTGAATTGG |
| JS2168 | 3C *PDC1* intergenic REV | GAAGCGGACCCAGACTTAAGC |
| JS2342 | Sir2 binding site 100bp∆ pCORE (FW) | GACTTACAAGCACACCTTTGAATTATTTTTGTTCTCTATGTCCTTACCATTAAGTTGATC |
| JS2343 | Sir2 binding site 100bp∆ pCORE (RV) | TATATAGCTATTCATCAATTGAAATATTCATTTTATAAGT GAGCTCGTTTTCGACACTGG |
| JS2444 | pCORE 100bp∆ replacement (FW) | GACTTACAAGCACACCTTTGAATTATTTTTGTTCTCTATGACTTATAAAATGAATATTTCAATTGATGAATAGCTATATA |
| JS2445 | pCORE 100bp∆ replacement (RV) | TATATAGCTATTCATCAATTGAAATATTCATTTTATAAGTCATAGAGAACAAAAATAATTCAAAGGTGTGCTTGTAAGTC |
| JS2494 | *RDT1* upstream  (-258 bp) (FW) | CGCGTTTAAAGACTTACAAGCAC |
| JS2496 | *RDT1* upstream  (-44 bp) (RV) | TTAAATACATGCTGCAGTTTTCG |
| JS2517 | *RDT1* upstream  (-19 bp) (FW) | AAAACTGCAGCATGTATTTAATCG |
| JS2518 | *RDT1* downstream (+19 bp) (RV) | TGCTTTCGATTATTTCTGGTTCT |
| JS2574 | Yalpha105F | GCCCACTTCTAAGCTGATTTCAATCTCTCC |
| JS2575 | MATdist-4R | CCTGTTCTTAGCTTGTACCAGAGG |
| JS2583 | *RDT1*-13xMyc_FW | AATTCTATTTGTCCAGCAATCCGGCGCAAAGAAGACTACCGGATCCCCGGGTTAATTAA |
| JS2584 | *RDT1*-13xMyc_RV | TTTCGATTATTTCTGGTTCTAGAAATTTTTCAATACCCTGAATTCGAGCTCGTTTAAAC |
| JS2585 | *RDT1* detection | TCTATTTGTCCAGCAATCCG |
| JS2656 | 3C *Hin*dIII *HML* | TTCCGAAAACCACGACGAACCAG |
| JS2658 | 3C *Hin*dIII *HMR* | ATGGGTCATTCTAGGTCATTCTAC |
| JS2665 | *SCR1* ORF (33 bp) (FW) | CGTTGAGAATTCTGGCCGAG |
| JS2666 | *SCR1* ORF (468 bp) (RV) | GTAAATCCTGATGGCACCGC |
| JS2669 | KanC3 | CCTCGACATCATCTGCCCAGAT |
| JS2703 | *HO* cut-site 600 bp (FW) | TTGGATCTTAACAAACCGTAAAGGT |
| JS2704 | *HO* cut-site 600 bp (RV) | GGTAACTAGCAAACAAAGGAAAGTCA |
| JS2712 | *MAT*a detection (FW) | TTGCAACAACTTCTTCTCCTCA |
| JS2715 | Alpha2 ORF_FW | TTGGTTTGCAAAGAACATCG |
| JS2716 | Alpha2 ORF_RV | CTTCTTTGCCAGAGGCTCAC |
| JS2777 | R1S/R1L ncRNA (FW) | CGTGCAAACAGTATTCCGGC |
| JS2778 | R1L/R1S ncRNA (RV) | GCGCTGGTTGTTATTGGCAA |
| JS3097 | *HMR* (FW) PCR | TGTGGCATTACTCCACTTCAAGTAAG |
| JS3098 | *HMR* (RV) real-time PCR | TCCGTCACCACGTACTTCAG |
| JS3100 | *HMR* (RV) PCR | TCGCAGTAGAAAGACATATTTCTC |
| JS3101 | *HML* (FW) PCR | TCTCTGCTCGCTGAAGAATGGC |
| JS3103 | *HML* (RV) PCR | ATGGAACACAGAAAAGAGCAGTG |
